# Relationship between nurses' resilience and depression, anxiety and stress during the 2021 COVID‐19 outbreak in Taiwan

**DOI:** 10.1002/nop2.1411

**Published:** 2022-10-26

**Authors:** Chiu‐Feng Wu, Tzu‐Hung Liu, Chu‐Hsuan Cheng, Kai‐Yen Chang

**Affiliations:** ^1^ Department of Nursing Taipei Tzu Chi Hospital, Buddhist Tzu Chi Medical Foundation New Taipei City Taiwan; ^2^ Department of Family Medicine Taipei Tzu Chi Hospital, Buddhist Tzu Chi Medical Foundation New Taipei City Taiwan; ^3^ School of Medicine Tzu Chi University Hualien Taiwan

**Keywords:** anxiety, COVID‐19, depression, nurse, resilience, work‐related stress

## Abstract

**Aim:**

The COVID‐19 outbreak in Taiwan had a significant impact on medical services. These changes posed a threat to nurses' mental health. Resilience may protect nurses from the psychological impact of COVID‐19. This study aimed to understand nurses' resilience and its relationship with nurses' characteristics (life and work situations) and mental health (depression, anxiety and stress) during the outbreak.

**Design:**

A cross‐sectional study.

**Methods:**

This study surveyed the nurses at a hospital from 9 August 2021, to 20 August 2021. The content of the questionnaire included nurses' characteristics, resilience and mental health.

**Results:**

There was an association between higher resilience and lower mental health problems. We also found that some nurses' characteristics were positively correlated with mental health problems.

**Conclusion:**

Some nurses' life and work situations predicted high levels of mental health problems during the pandemic. Additionally, higher levels of resilience were associated with lower levels of mental health problems.

## 1 INTRODUCTION

Coronavirus disease 2019 (COVID‐19) is an infectious disease caused by severe acute respiratory syndrome coronavirus 2 (SARS‐CoV‐2), which is transmitted mainly by contact with droplets containing the virus in the air; due to this ease of transmission, the disease quickly became a pandemic (Ciotti et al., 2020). In May 2021, COVID‐19 spread rapidly throughout Taiwan. This outbreak resulted in approximately 16,600 local cases and killed approximately 800 (Centers for Disease Control, Ministry of Health and Welfare, 2021).

The pandemic brought significant changes to the pattern of medical services in Taiwan. During the outbreak, nurse practitioners added more protective measures to avoid infection, adapted to large numbers of patients (Chen et al., 2020; Feng et al., 2020; Su et al., 2020) were deployed throughout different units within the hospital (Liang et al., 2021; Su et al., 2020), supported enhanced isolation hotels (Tsai et al., 2021), community screening stations and community vaccination stations. Nurses lived with the fear that they might contract COVID‐19 and transmit it to their families and that people might reject or discriminate against them or their families because of their profession (Sampaio et al., 2020; Su et al., 2020; Zhang et al., 2022). These dramatic changes left them stressed and at risk for mental health problems. Nurses were prone to experience depression, anxiety (Sampaio et al., 2020; Su et al., 2020; Zhang et al., 2022), sleep problems (Zhang et al., 2022) and serious symptoms, such as posttraumatic stress disorder (Galli et al., 2020). These problems can lead to mental disorders or burnout and ultimately cause nurses to quit their jobs (Labrague & Santos, 2021). It is crucial to understand whether there are protective factors that can mitigate the psychological impact of the ongoing pandemic and promote nurses' mental health. Building resilience is considered one of the approaches to reduce the psychological impact of adversity on nurses (Alhawatmeh et al., 2021; Labrague & Santos, 2021).

Resilience is the ability to remain positive and adapt to adversity (Schetter & Dolbier, 2011). It can be strengthened by education or training (Adibi Larijani & Garmaroudi, 2018). Some studies show that resilience can be regarded as a protective factor of mental health (Thompson & Dobbins, 2018) and can reduce the adverse impact of trauma on mental health (Brassington & Lomas, 2020). Additionally, research shows that resilience can modulate the relationship between stress and negative emotions and reduce the impact of stress (Manomenidis et al., 2019).

This study aimed to understand nurses' resilience and its relationship with nurses' demographic characteristics, life and work situations, depression, anxiety and stress during the COVID‐19 outbreak. The study also aimed to provide suggestions for future hospital work policies and educational interventions when facing emerging infectious diseases.

## 2 METHODS

This study was designed as a cross‐sectional study, and the target population was surveyed with an online questionnaire. The sample was 930 nurses who worked in a teaching hospital located in northern Taiwan. Responses were collected from 9 August 2021, to 20 August 2021.

### 2.1 Questionnaire

The content of the survey included questions about nurses' characteristics, resilience, depression, anxiety and stress.

#### 2.1.1 Nurse characteristics

Demographic characteristics of interest included age, years of nursing experience, clinical ladder level, gender, marital status and religious beliefs. There were six clinical ladder levels: (1) N: newly graduated Registered Nurses; (2) N1: nurses who had more than one year of clinical experience; (3) N2: nurses who had been N1 for more than one year and qualified for critical care; (4) N3: nurses who had been N2 for more than one year and qualified for teaching and holistic care; (5) N4: nurses who had been N3 for more than one year and qualified for research and specialty care; and (6) NP: nurses who had passed the board certification examination for nurse practitioners. Life and work situations during the outbreak included vaccination status, caring for COVID‐19 patients before the local outbreak, caring for family members (elders or children), concern about family members becoming infected, perceived risk of being infected by COVID‐19, family members experiencing discrimination due to their occupation, housing problems during the outbreak (e.g. needing to live away from home), caring for COVID‐19 patients during the outbreak, being transferred to another work unit during the outbreak, shift situation and supporting community screening or vaccination programs during the outbreak.

#### 2.1.2 Resilience

Resilience was measured using a scale developed by Hsiao et al. (2019), and the scale was validated among a population of Taiwanese nurses. The resilience scale has 10 items rated on a 5‐point Likert scale. Participants were scored from 1 (strongly disagree) to 5 (strongly agree) based on the degree of conformity between the situation described in each item and their own situation. A higher score indicated a higher level of resilience. Hsiao et al. (2019) reported a goodness‐of‐fit index of 0.973 and a Cronbach's α of 0.91, showing good fit and internal consistency.

#### 2.1.3 Depression, anxiety and stress

Depression, anxiety and stress were measured by the 21‐item Depression Anxiety and Stress Scale (DASS‐21), which was developed by Lovibond and Lovibond (1995) based on the theoretical model of Clark and Watson (1991). There are three dimensions (depression, anxiety and stress) in this scale, and each dimension contains seven items. Participants were scored from 0 (did not apply to me at all) to 3 (applied to me very much or most of the time) according to their situation during the previous week. Higher scores on the DASS‐21 indicate a more severe psychological state (depression, anxiety or stress). The five levels of severity of depression are normal (0–9), mild (10–13), moderate (14–20), severe (21–27) and extremely severe (28+). The five levels of anxiety are normal (0–7), mild (8–9), moderate (10–14), severe (15–19) and extremely severe (20+). The five levels of stress were normal (0–14), mild (15–18), moderate (19–25), severe (26–33) and extremely severe (34+). The internal consistency reliability (Cronbach's α) of the original version of the DASS‐21 was 0.87–0.94 (Antony et al., 1998), whereas the Cronbach's α of the Chinese version of the DASS‐21 used in this study was 0.94, indicating excellent internal consistency of the scale.

#### 2.1.4 Data analysis

The relationships among age, years of nursing experience and scaled score were analysed by correlation coefficient analysis. Differences in scale scores between variables were analysed using Student's t‐test or one‐way analysis of variance. Multiple regression analysis was used to adjust and determine which characteristics predicted depression, anxiety and stress. The statistical software used in this study was R 4.1.1 (R Core Team, 2021).

#### 2.1.5 Ethical considerations

This study was approved by the Institutional Review Board of Taipei Tzu Chi Hospital (IRB No. 10‐XD‐109). The approved study conduction time was from 1 August 2021, to 1 August 2022. This study was conducted with an anonymous online questionnaire that was emailed to all nurses working in the hospital. The content of the email and the first page of the questionnaire were informed consent messages that the nurses had to read and agree to before filling out the questionnaire. The authors of the resilience scale approved its use in this study, and the DASS‐21 scale is a free resource (Psychology Foundation of Australia, 2018).

## 3 RESULTS

The questionnaire was sent to 930 nurses by email, and a total of 600 valid responses were collected for a response rate of 64.5%.

Descriptive statistics are shown in Table 1. The mean age of the participants was 31.19 ± 9.07 years. The mean years of nursing experience were 8.81 ± 7.98. Nearly half of the participants were at the N2 clinical ladder level (47.50%). Approximately, one‐quarter of the participants worked in the medical ward (26.17%). Most were unmarried (74.17%) and had graduated from university (68.43%), received one dose of COVID‐19 vaccine (91.50%), did not care for COVID‐19 patients before the outbreak (79.33%) and did not need to take care of other family members (67.83%). Most of the participants worried that their family members would become infected (86.83%). Most also perceived a high risk of infection (76.83%), noted that their family members did not experience discrimination (83.83%), had no housing problems (76.17%), did not have to care for COVID‐19 patients (57.17%), were not transferred to another unit to work (78.17%), experienced no shift situation (51.33%) and were not required to support community screening or vaccination (85.67%). The participants had a mean resilience score of 37.23 ± 6.31, a mean depression score of 6.93 ± 7.11, a mean anxiety score of 6.57 ± 6.36 and a mean stress score of 10.90 ± 7.79. The mean resilience score of our participants was slightly lower than that of the group in the validation study (38.44 ± 5.77) (Hsiao et al., 2019). The DASS‐21 results, according to the cut‐off scores for conventional severity labels, indicated that 33.7% of participants had depressive symptoms, 36.8% of participants had anxiety, and 38.0% of participants felt stressed.

**TABLE 1:** Characteristics, resilience and DASS‐21 of nurse participants in this study (*N* = 600)

Variables	Mean	SD
Age	31.19	9.07
Years of nursing experience	8.81	7.98
Clinical ladder levels (Levels of Nursing Credentials)^a^	N: 97 (16.17%), N1: 101 (16.83%), N2: 285 (47.50%), N3: 61 (10.17%), N4: 20 (3.33%), NP: 36 (6.00%)	
Gender	Female: 569 (94.83%), Male:31 (5.17%)	
Marital status	Unmarried: 445 (74.17%), married: 155 (25.83%)	
Have religious beliefs	No: 370 (61.67%); Yes: 230 (38.33%)	
Have been vaccinated	No: 7 (1.17%); One dose: 549 (91.50%); Two doses: 44 (7.33%)	
Caring for COVID‐19 patients before the local outbreak	No: 476 (79.33%); Yes: 124 (20.67%)	
Need to take care of family members (elders or kids)	No: 407 (67.83%); Yes, alone: 24 (12.44%); Yes, with another family member: 169 (87.56%)	
Worried about family members getting infected	No: 79 (13.17%); Yes: 521 (86.83%)	
Perceived risk of being infected by COVID‐19	Not likely to happen: 42 (7.00%); Possible: 461 (76.83%); Very likely to happen: 97 (16.17%)	
Had family members been discriminated against because of your occupation?	No: 503 (83.83%); Yes: 97 (16.17%)	
Had housing problems during the outbreak	No: 457 (76.17%); Need to live in separate rooms: 58 (9.67%); Need to apply for staff quarters: 24 (4.00%); Need to live outside: 47 (7.83%); Other housing problem: 14 (2.33%)	
Caring for COVID‐19 patients during the local outbreak	No: 343 (57.17%); Not in a dedicated COVID‐19 unit, but still had to care for COVID‐19 patients: 49 (8.17%); Yes, in a dedicated COVID‐19 wards: 184 (30.67%); Yes, in a quarantine hotel: 24 (4.00%)	
Transfer to another unit to work during the local outbreak	No: 469 (78.17%); Voluntary: 94 (15.67%); Assigned: 37 (6.17%)	
Shift status during the pandemic	Fixed shift: 308 (51.33%); Three‐shifts: 282 (47.00%); Other type of shifts: 10 (1.67%)	
Have supported community screening or vaccination during the local outbreak	No: 514 (85.67%); Yes: 86 (14.33%)	
Resilience	37.23	6.31
DASS‐21		
Depression subscale score	6.93	7.11
Anxiety subscale score	6.57	6.36
Stress subscale score	10.90	7.79

^a^ N: new graduate Registered Nurses; N1: nurses who have more than one year of clinical experience; N2: nurses who have been N1 for more than one year and qualified for critical care; N3: nurses who have been N2 for more than one year and qualified for teaching and holistic care; N4: nurses who have been N3 for more than one year and qualified for research and specialty care; NP: nurses who passed the board certification examination of nurse practitioners.

### 3.1 Results of correlation coefficient analysis

The results of the correlation coefficient analysis are shown in Table 2. Participants' age (*R* = 0.27, *p* < .001) and years of nursing experience (*R* = 0.26, *p* < .001) both had a weak but positive correlation with the resilience score, indicating that the older or more experienced nurses were, the more resilient they were. Participants' age also had a very weak, negative correlation with depression (*R* = −0.093, *p* < .05) and anxiety (*R* = −0.091, *p* < .05) scores. When examining the relationship between resilience and depression, anxiety or stress, the resilience score had a moderate negative correlation with the depression score (*R* = −0.43, *p* < .001), anxiety score (*R* = −0.31, *p* < .001) and stress score (*R* = −0.36, *p* < .001). This indicates that higher resilience scores could predict lower scores for depression, anxiety and stress.

**TABLE 2:** Results of correlation coefficient analysis (*N* = 600)

Variables			Age	Years of nursing experience	Resilience total score	DASS‐21		
						Depression subscale score	Anxiety subscale score	Stress subscale score
Age		*R* ^2^	1					
		*p*	0					
Years of nursing experience		*R* ^2^	0.92**	1				
		*p*	<.001	0				
Resilience total score		*R* ^2^	0.27**	0.26**	1			
		*p*	<.001	<.001	0			
DASS‐21	Depression subscale score	*R* ^2^	−0.093*	−0.062	−0.43**	1		
		*p*	.022	.13	<.001	0		
	Anxiety subscale score	*R* ^2^	−0.091*	−0.051	−0.31**	0.78**	1	
		*p*	.026	.21	<.001	<.001	0	
	Stress subscale score	*R* ^2^	−0.08	−0.034	−0.36**	0.82**	0.8**	1
		*p*	.05	.41	<.001	<.001	<.001	0

* *p* < .05.

** *p* < .001.

### 3.2 Results of multiple regression analysis

Participants' age, gender and other characteristics were included in the regression model to analyse which characteristics could predict depression, anxiety and stress. The results are shown in Table 3.

**TABLE 3:** Multiple regression analysis on the predictor of scores for each subscale of DASS‐21 (*N* = 600)

	Depression	Anxiety	Stress
Variables	Beta (SE)	Beta (SE)	Beta (SE)
(Intercept)	22.37 (2.14)**	15.03 (1.96)**	23.99 (2.37)**
Age	0.00 (0.04)	0.00 (0.03)	−0.04 (0.04)
Gender			
Female	(reference)	(reference)	(reference)
Male	1.64 (1.20)	−0.41 (1.11)	0.36 (1.33)
Clinical ladder levels (Levels of Nursing Credentials)^a^			
N	(reference)	–	(reference)
N1	−0.63 (0.93)	–	−0.39 (1.03)
N2	1.15 (0.84)	–	1.65 (0.94)
N3	2.42 (1.15)*	–	2.84 (1.28)*
N4	5.33 (1.75)*	–	6.33 (1.95)**
NP	0.90 (1.42)	–	1.91 (1.57)
Marital status			
Unmarried	(reference)	–	–
Married	−0.73 (0.71)	–	–
Caring for COVID‐19 patients before the local outbreak			
No	(reference)	(reference)	(reference)
Yes	0.96 (0.68)	0.76 (0.62)	1.18 (0.82)
Worried about family members getting infected			
No	–	(reference)	(reference)
Yes	–	0.97 (0.75)	1.76 (0.89)*
Perceived risk of being infected by COVID‐19			
Not likely	(reference)	(reference)	(reference)
Possible	0.50 (1.03)	0.41 (0.99)	−0.46 (1.18)
Very likely	2.00 (1.19)	2.26 (1.15)*	1.78 (1.36)
Had family members been discriminated against because of your occupation?			
No	(reference)	(reference)	(reference)
Yes	1.76 (0.71)*	1.71 (0.68)*	2.93 (0.80)**
Had housing problems during the outbreak			
No	–	(reference)	(reference)
Need to live in separate rooms	–	0.65 (0.85)	−0.44 (1.00)
Need to apply for staff quarters	–	0.18 (1.26)	0.18 (1.50)
Need to live outside	–	2.22 (0.93)*	2.21 (1.11)*
Other housing problem	–	−0.47 (1.64)	0.35 (1.93)
Caring for COVID‐19 patients during the local outbreak			
No	–	–	(reference)
Not in a dedicated COVID‐19 unit, but still had to care for COVID‐19 patients	–	–	1.17 (1.09)
Yes, in a dedicated COVID‐19 wards	–	–	−1.11 (0.73)
Yes, in a quarantine hotel	–	–	2.95 (1.50)*
Shift status during the pandemic			
Fixed shift	(reference)	(reference)	(reference)
Three shift	0.74 (0.58)	0.67 (0.52)	1.05 (0.63)
Other types of shifts	2.84 (2.11)	1.75 (1.93)	1.50 (2.32)
Have supported community screening or vaccination during the local outbreak			
No	(reference)	–	–
Yes	−1.00 (0.79)	–	–
Resilience total score	−0.47 (0.04)**	−0.29 (0.04)**	−0.42 (0.05)**
Goodness of fit	Adjust *R* ^2^ = 0.22; *F* = 11.41; *p* < .001**	Adjusted *R* ^2^ = 0.13; *F* = 7.32; *p* < .001**	Adjusted *R* ^2^ = 0.20; *F* = 7.81; *p* < .001**

*Note*: Perform regression analysis only on the variables which significantly affect DASS‐21 scores as predictors and adjust by nurses' age and gender.

*p < .05; **p < .001.

^a^ N: new graduate Registered Nurses; (2) N1: nurses who have more than one year of clinical experience; (3) N2: nurses who have been N1 for more than one year and qualified for critical care; (4) N3: nurses who have been N2 for more than one year and qualified for teaching and holistic care; (5) N4: nurses who have been N3 for more than one year and qualified for research and specialty care; (6) NP: nurses who passed the board certification examination of nurse practitioners.

The coefficient of determination of the Model (*R* ^2^) for depression was 0.22 (*p* < .001). Participants whose clinical ladder levels were N3 (*β* = 2.42, *p* < .05) or N4 (*β* = 5.33, *p* < .01) had higher depression scores than those whose levels were N. Participants whose family members had experienced discrimination had higher depression scores (*β* = 1.76, *p* < .05). Participants with higher resilience scores had lower depression scores (*β* = −0.47, *p* < .001).

The coefficient of determination of the Model (*R* ^2^) for anxiety was 0.13 (*p* < .001). The participants who perceived a high risk of being infected by COVID‐19 had higher anxiety scores than those who did not feel that they were at risk of infection (*β* = 2.26, *p* < .05). The participants whose family members had experienced discrimination had higher anxiety scores (*β* = −1.71, *p* < .05). Participants who needed to live away from home during the outbreak had higher anxiety levels than those not affected in this manner (*β* = 2.22, *p* < .05). Participants with higher resilience scores had lower anxiety scores (*β* = −0.29, *p* < .001).

The coefficient of determination of the Model (*R* ^2^) for stress was 0.20 (*p* < .001). Participants whose clinical ladder levels were N3 (*β* = 2.84, *p* < .05) or N4 (*β* = 6.33, *p* < .01) had higher stress scores than those whose levels were N. Participants who worried that family members might become infected had higher stress scores than those who did not worry about it (*β* = 1.76, *p* < .05). Participants whose family members had experienced discrimination had higher stress scores (*β* = 2.93, *p* < .001). Participants who needed to live away from home during the outbreak had higher stress scores than those not affected in this manner (*β* = 2.21, *p* < .05). Participants who supported community screening or vaccination programs during the outbreak had higher stress than those who did not (*β* = 2.95, *p* < .05). Participants with higher resilience scores had lower stress scores (*β* = −0.42, *p* < .001).

## 4 DISCUSSION

The primary objective of this study was to assess nurses' resilience and its relationship with nurses' demographics, living and working conditions, and their depression, anxiety and stress during the COVID‐19 outbreak.

First, we found slightly lower resilience and a relatively high proportion of mental health problems in our participants during the pandemic. The resilience score of our participants was slightly lower than that of the group in the validation study (Hsiao et al., 2019). McAllister and McKinnon (2009) suggested that individual resilience would be changed in different contexts, and Cooper et al. (2020) reported in their concept analysis that nurses' resilience is related to social support, self‐efficacy, work–life balance/self‐care, humour, optimism and being realistic. The results of our study indicate that participants were experiencing changes in their lives and work, which might have lowered their resilience. We also found that 33%–40% of our participants had problems with depression, anxiety or stress. Our results are consistent with previous research (Sampaio et al., 2020; Zhang et al., 2022). In the study by Sampaio et al. (2020), nurses experienced higher levels of depression, anxiety and stress problems even before the real COVID‐19 outbreak in their country. In the study by Zhang et al. (2022), 46% of frontline nurses in Wuhan had depression problems, and 40% had anxiety problems.

Second, the relationship between nurses' resilience and characteristics was examined, and the older the nurses were and the greater their years of nursing experience, the more resilient they were during the outbreak. These results matched the findings of previous research (Ang et al., 2018; Hsiao et al., 2019; Öksüz et al., 2019).

Next, the relationships between nurses' characteristics and depression, anxiety or stress were also analysed. Nurses whose clinical ladder levels were N3 or N4 had more depression than those at the N level, and nurses at the N4 level had more stress than those at the N level. Clinical ladder levels generally indicate the job training status of nurses. The higher the clinical ladder level was, the better the nursing skills and knowledge. These findings of the relationship between clinical ladder levels and depression and stress were not consistent with other studies (Huang et al., 2021; Murat et al., 2021). Murat et al. (2021) surveyed Australian nurses from May 2020 to June 2020. They found that nurses with fewer years of nursing experience and lower confidence in their nursing skills had higher stress levels than others. The reason why the findings from this study differ from other studies may be because nurses whose clinical ladder levels were N3 (mean years of nursing experience: 13.54 ± 6.07) or N4 (mean years of nursing experience: 22.70 ± 9.49) had to take on heavier clinical duties during the outbreak than nurses at entry level or with fewer years of nursing experience.

This study found that nurses who worried they might contract and spread COVID‐19 to their families had higher anxiety levels than nurses who did not. This is similar to the findings of Han et al. (2020) and Sampaio et al. (2020). Sampaio et al. (2020) reported that concern about being infected and spreading COVID‐19 to their families were the primary sources of depression, anxiety and stress among nurses.

Some research has suggested that nurses or other medical professionals experienced discrimination during the COVID‐19 pandemic (Hong et al., 2021; Labrague et al., 2021) and that this affected their mental health and increased their intention to leave their jobs. More seriously, this discrimination extended to their family members (Jamieson et al., 2021; Simeone et al., 2021). According to Jamieson et al. (2021) and Simeone et al. (2021), since nursing was considered a high‐risk occupation in terms of COVID‐19 infection, nurses' family members were also considered to be at high risk of contracting and spreading the virus. In our study, some nurses reported that their family members were discriminated against and perceived to have a high risk of COVID‐19 infection and spreading the disease, which was associated with elevated levels of depression, anxiety and stress among the nurses.

In this study, nurses who could not live in their own homes during the COVID‐19 outbreak had higher levels of anxiety and stress than other nurses. These findings were similar to the results of Sampaio et al. (2020). These nurses were afraid to spread COVID‐19 to their family members, so they chose not to live at home during the outbreak. Some nurses in this study chose to live away from home because they did not have a dormitory provided by the hospital. Labrague and Santos (2021) reported that nurses who lacked organizational or community support had higher anxiety levels than others. In a survey following this study in our hospital, some nurses who had to live away from home responded that the residential support provided by the hospital did not meet their needs, and they were forced to find alternative accommodations during the COVID‐19 outbreak. This might explain why they had higher anxiety and stress levels.

This study found that nurses supporting the enhanced quarantine hotel (a housing facility for COVID‐19 patients with mild symptoms) had higher stress levels than other nurses. This could be explained by an increased risk of infection (Sampaio et al., 2020) or an unfamiliar work environment (Labrague & Santos, 2021).

Finally, we analysed the relationship between resilience and depression, anxiety or stress. In this study, resilience had a protective effect on nurses' depression, anxiety and stress levels, similar to the findings of prior studies (Alhawatmeh et al., 2021; Labrague & Santos, 2021). Labrague and Santos (2021) surveyed frontline nurses and found that nurses with more social support, more organizational support or high levels of resilience had lower anxiety levels. Manomenidis et al. (2019) described that resilience could adjust for the effect of stressors and negative emotions. Alhawatmeh et al. (2021) surveyed Registered Nurses and found that resilience was a mediating variable. Resilience partially mediates the association between Registered Nurses' perceived stress and their quality of life. In our study, we further conducted regression analysis tests to examine the mediating effect of resilience and found that resilience partially buffered the effect of perceived stress on depression, which supported that resilience had a protective effect on the emotional status of nurses during the pandemic.

Based on the findings of this study, the senior management team at hospitals may be able to reduce nurses' experiences of depression, anxiety and stress during the pandemic by providing organizational support and classes that aim to enhance nurses' resilience.

### 4.1 Limitations

There were several limitations to this study. First, this study involved a self‐report questionnaire, and participants' answers might not represent their actual situations. Second, the questionnaire was anonymous and sent to each nurse by email, so it could have been missed. However, valid questionnaires were returned by more than 60% of the nurses. Third, all of the participants were from the same hospital, so the results might not be applicable to nurses at different hospitals. However, the study hospital was one of the largest, where most COVID‐19 patients in Taiwan were hospitalized. Additionally, this study had a relatively large number of participants, so the survey results could have significant value in Taiwan.

## 5 CONCLUSION

In this study, some of the nurses' life and work situations during the COVID‐19 outbreak in Taiwan were associated with higher levels of depression, anxiety and stress. Nurses with high levels of resilience appeared to be protected against depression, anxiety and stress. This study suggests that the senior management team at hospitals may reduce nurses' experiences of depression, anxiety and stress during the pandemic by providing organizational support and classes that aim to enhance nurses' resilience.

### CLINICAL RESOURCES

1. Keep health workers safe to keep patients safe: https://www.who.int/news/item/17‐09‐2020‐keep‐health‐workers‐safe‐to‐keep‐patients‐safe‐who
2. Help and support for healthcare workers—coronavirus (COVID‐19): https://www.dhhs.vic.gov.au/help‐and‐support‐healthcare‐workers‐coronavirus‐covid‐19
3. Free Tools and Apps to Support the Mental Health and Resilience of All Nurses: https://www.nursingworld.org/practice‐policy/work‐environment/health‐safety/disaster‐preparedness/coronavirus/what‐you‐need‐to‐know/the‐well‐being‐initiative/

## AUTHOR CONTRIBUTIONS

Chiu‐Feng Wu designed the whole study and research progress control. Kai‐Yen Chang was responsible for data collecting, research progress control and second revision of draft. Tzu‐Hung Liu was responsible for the design of the form and the final revision. Chu‐Hsuan Cheng was responsible for the data analysis of the study and writing the first draft of the article.

## FUNDING INFORMATION

This research was found by Taipei Tzu Chi hospital holistic nursing research team (project number: TCRD‐TPE‐109‐RT‐9). The funders of the study had no role in study design, data collection, data analysis, data interpretation or writing of the report. The corresponding author had full access to all the data in the study and had final responsibility for the decision to submit for publication.

## CONFLICTS OF INTEREST

The authors report no actual or potential conflicts of interest.

## ETHICAL APPROVAL

This study was approved by the Institutional Review Board of Taipei Tzu Chi hospital (IRB No. 10‐XD‐109). Hsiao and her team approved the usage of their resilience scale in this study, and the DASS‐21 scale is a free resource and could be used in this study (Psychology Foundation of Australia, 2018).

## Data Availability

The data that support the findings of this study are available from the corresponding author upon reasonable request.
